# Supplementary material for: Locus-specific histone deacetylation using a synthetic CRISPR-Cas9-based HDAC
Source: Nat Commun. 2017 May 12;8:15315. doi: 10.1038/ncomms15315 (PMC5437308; doi:10.1038/ncomms15315)
Supplement: Supplementary Information — Supplementary Figure and Supplementary Tables [file ncomms15315-s1.pdf]

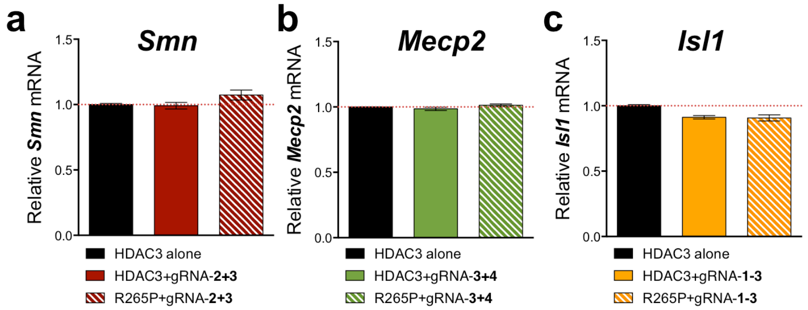


**Supplementary Fig. 1. Multiplexing gRNAs with dCas9-HDAC3 does not result in synergistic regulation of gene expression.** 48 hr co-transfection of a) *Smn* gRNAs 2 and 3; b) *Mecp2* gRNAs 3 and 4; c) and *Isl1* gRNAs 1, 2, and 3, with either dCas9-HDAC3 or dCas9-HDAC3-R265P do not significantly alter the expression of each respective gene relative to dCas-HDAC3 alone. *Smn* (n= 5); *Mecp2* (n=6); *Isl1* (n=3) biological replicates; error bars, s.e.m.

| gRNA | Position (to TSS) | Sequence |
|---|---|---|
| Scrambled | N/A | ACATCGGCCGTTACACGCGT |
| *Smn1* gRNA-1 | +1 bp | TCAATGACGACTTCCGTCAT |
| *Smn1* gRNA-2 | -325 bp | CCAACCCATCGTCTAATAAT |
| *Smn1* gRNA-3 | -463 bp | TGGGATATCGAAATTCTATC |
| *Mecp2* gRNA-1 | +85 bp | GGTAAAACCCGTCCGGAAAA |
| *Mecp2* gRNA-2 | +13 bp | GGCGCGACGTCTGCCGTGCG |
| *Mecp2* gRNA-3 | -101 bp | AATTGAGGGCGTCACCGCTA |
| *Mecp2* gRNA-4 | -594 bp | AATGACAGACCGATCTCTTA |
| *Isl1* gRNA-1 | -30 bp | TTGGTCTGACGCAGCGCGCG |
| *Isl1* gRNA-2 | -186 bp | CCCAAGGAGCGACGTGGCCT |
| *Isl1* gRNA-3 | -533 bp | CTAGCAGCGCGCTACGCGTT |

**Supplementary Table 1:** Sequences used for the construction of gRNAs in this study and their genomic positions relative to the transcriptional start site (pink background= *Smn1*; green background= *Mecp2*; orange background= *Isl1*).

| Target Position | Forward Primer | Reverse Primer |
|---|---|---|
| -149 bp | CCCTGCCTAAACAGACAGGAA | CCACCCCTTGCTCTTTGTCG |
| -169 bp | GAATGGGGTCCGCCTCTTTT | CTTCATTGGTTGTGGAGCCC |
| -330 bp | TGTTGTTCAGAAATACCACACTATC | GCAGACTGCATCTCTTGTTTAG |
| -547 bp | AAAGCCTGGTGCCCATTTA | CACTCTGCCTGTCTTCCATAAC |
| -2 kb | TCCCAAAATTTCACAAGGTAGGAA | TGAGAACAAAGCAAGAAACAGCA |

**Supplementary Table 2:** Sequences of primers used for ChIP-qPCR.

| Sequence | Mismatches | Locus |
|---|---|---|
| AATTATGCGGGTCACCGCTATAG | 4MMs [5:6:8:10] | chr14:-87271721 |
| AATTCAGTGAGTCACCGCTTCAG | 4MMs [5:8:10:20] | chr13:-53144845 |
| AAGTGAGGGCGTCACCTCTCAAG | 3MMs [3:17:20] | chr15:+27716529 |
| AAAGGAGGGCCTGACCGCTAAGG | 4MMs [3:4:11:13] | chr11:-113094450 |
| AAGTGAGGGCATCACTGCTACAG | 3MMs [3:11:16] | chr9:+70628124 |
| AACTGAAGGCATGACCGCTATAG | 4MMs [3:7:11:13] | chr14:+15154210 |
| AATGGAGGGTGTCACCACCATAG | 4MMs [4:10:17:19] | chr14:-115873285 |
| AATTGAGGTTTTCACCTCTAAGG | 4MMs [9:10:11:17] | chr5:+145880576 |
| AAGTGAGGGGGTCACAGCTTGAG | 4MMs [3:10:16:20] | chr10:-121951495 |
| CATTGAAGGCCTCACAGCTAAAG | 4MMs [1:7:11:16] | chr16:-27200510 |
| ACTTGAGGACTTCACAGCTAGAG | 4MMs [2:9:11:16] | chr2:-88386315 |
| AATTTAGGGCTTCACCAGTAGAG | 4MMs [5:11:17:18] | chr2:+35718710 |
| AGTTGAGGGCCTCCCCGCTGGGG | 4MMs [2:11:14:20] | chr6:-70719968 |
| AATTGATGACGTCAGAGCTAGAG | 4MMs [7:9:15:16] | chr3:-108973854 |
| AATTGAGGGCTTTACCATTAAAG | 4MMs [11:13:17:18] | chr14:+110352784 |
| AATTGAGGGCTTCAAAGCTCTGG | 4MMs [11:15:16:20] | chr16:-25068499 |
| AATTGAGGGAGTCACAGGGAAGG | 4MMs [10:16:18:19] | chr12:+9173046 |

**Supplementary Table 3:** Sequences of potential genome-wide off-target sites for *Mecp2* gRNA-3, their number of mismatches (MM) and genomic locations (www.crispr.mit.edu).
